# Sevoflurane Effects on Neuronal Energy Metabolism Correlate with Activity States While Mitochondrial Function Remains Intact

**DOI:** 10.3390/ijms23063037

**Published:** 2022-03-11

**Authors:** Mathilde Maechler, Jörg Rösner, Iwona Wallach, Joerg R. P. Geiger, Claudia Spies, Agustin Liotta, Nikolaus Berndt

**Affiliations:** 1 Institute of Neurophysiology, Charité-Universitätsmedizin Berlin, Corporate Member of Freie Universität Berlin and Humboldt-Universität zu Berlin, 10117 Berlin, Germany; mathilde.maechler@charite.de (M.M.); joerg.roesner@charite.de (J.R.); joerg.geiger@charite.de (J.R.P.G.); agustin.liotta@charite.de (A.L.); 2 Institute of Biochemistry, Charité-Universitätsmedizin Berlin, Corporate Member of Freie Universität Berlin and Humboldt-Universität zu Berlin, 10117 Berlin, Germany; iwona.wallach@charite.de; 3 Department of Anesthesiology and Intensive Care, Charité-Universitätsmedizin Berlin, Corporate Member of Freie Universität Berlin and Humboldt-Universität zu Berlin, 10117 Berlin, Germany; claudia.spies@charite.de; 4 Neuroscience Research Center, Charité-Universitätsmedizin Berlin, Corporate Member of Freie Universität Berlin and Humboldt-Universität zu Berlin, 10117 Berlin, Germany; 5 Institute of Computer-assisted Cardiovascular Medicine, Charité-Universitätsmedizin Berlin, Corporate Member of Freie Universität Berlin and Humboldt-Universität zu Berlin, 13353 Berlin, Germany; 6 Berlin Institute of Health at Charité-Universitätsmedizin Berlin, 10117 Berlin, Germany

**Keywords:** sevoflurane, neuron, metabolism

## Abstract

During general anesthesia, alterations in neuronal metabolism may induce neurotoxicity and/or neuroprotection depending on the dose and type of the applied anesthetic. In this study, we investigate the effects of clinically relevant concentrations of sevoflurane (2% and 4%, i.e., 1 and 2 MAC) on different activity states in hippocampal slices of young Wistar rats. We combine electrophysiological recordings, partial tissue oxygen (p_ti_O_2_) measurements, and flavin adenine dinucleotide (FAD) imaging with computational modeling. Sevoflurane minimally decreased the cerebral metabolic rate of oxygen (CMRO_2_) while decreasing synaptic transmission in naive slices. During pharmacologically induced gamma oscillations, sevoflurane impaired network activity, thereby decreasing CMRO_2_. During stimulus-induced neuronal activation, sevoflurane decreased CMRO_2_ and excitability while basal metabolism remained constant. In this line, stimulus-induced FAD transients decreased without changes in basal mitochondrial redox state. Integration of experimental data and computer modeling revealed no evidence for a direct effect of sevoflurane on key enzymes of the citric acid cycle or oxidative phosphorylation. Clinically relevant concentrations of sevoflurane generated a decent decrease in energy metabolism, which was proportional to the present neuronal activity. Mitochondrial function remained intact under sevoflurane, suggesting a better metabolic profile than isoflurane or propofol.

## 1. Introduction

The discovery and refining of general anesthesia techniques represent a milestone in contemporary medicine allowing major advances in surgical therapies and intensive care. Anesthetics induce a state generally described as pharmacological coma although different dynamic brain states occur depending on the clinical situation, type of anesthetic, and the applied dose [1]. Concerning possible neuroprotective or neurotoxic effects on neurons, the available research is still controversial [2]. In particular, deep anesthesia (characterized with electroencephalography) has been associated with postoperative neurological complications, such as postoperative delirium, long-term cognitive decline, stroke, and increased mortality in intensive care patients [3,4,5]. On the other hand, the brain’s metabolism might decrease proportionally to the applied anesthetic dose [1,6], and titration to reach deep anesthesia is empirically used to lower brain metabolism and achieve neuroprotection during neurosurgery or in severe brain conditions, such as status epilepticus or intracranial hypertension [7,8].

Neurons consume more than 50% of their produced adenosine triphosphate (ATP) to actively maintain transmembrane ion gradients needed for reliable action potential generation and neurotransmission [9,10]. Thus, an anesthetic-induced decrease in neuronal metabolism mainly results indirectly from inhibition of energy-intensive processes involved in action potential generation and neurotransmission. Additionally, direct mitochondrial dysfunction has been described for almost all available anesthetics and represents a potential mechanism of neurotoxicity contributing to perioperative brain dysfunction and long-term neurocognitive deficits [11,12,13,14,15,16,17].

The halogenated ether sevoflurane exerts multiple effects on neurotransmission [18] including enhancement of GABA_A_-mediated inhibition [19], blockade of glutamatergic transmission [20,21], decreased presynaptic vesicle exocytosis [22], and activation of two-pore-domain potassium channels [23,24,25].

Concerning the effects of sevoflurane on neurometabolism, the available data are conflicting since neuroprotective and neurotoxic effects have been described. Sevoflurane preconditioning has been shown to promote neuroprotection after ischemia and oxygen/glucose deprivation [26]. Potential protective mechanisms included mitochondrial K^+^-ATP channel opening [27], inhibition of mitochondrial permeability transition pore opening [28], and apoptosis inhibition [29]. Conversely, mitochondrial impairment was induced by sevoflurane in different neonatal, aged, and Alzheimer’s disease animal models. Among others, mechanisms involved were mitophagy inhibition [30], lower oxidative phosphorylation [31], iron/glucose metabolism-derangement [32], and mitochondrial depolarization via complex-V inhibition [33]. 

Since sevoflurane has become one of the most popular volatile anesthetics in the clinical routine, further studies are necessary to better understand whether changes in neurometabolism are secondary to decreased synaptic activity or the consequence of a primary impairment of mitochondrial processes needed for energy homeostasis.

In this study, we investigated the effects of sevoflurane on neuronal metabolism in acute brain slices of young adult Wistar rats. For this purpose, we combined electrophysiological recordings, partial tissue oxygen pressure (p_ti_O_2_) measurements, and flavin adenine dinucleotide (FAD) imaging with computational modeling.

## 2. Results

### 2.1. Effects of Sevoflurane on Cerebral Metabolic Rate of Oxygen (CMRO_2_) and Synaptic Activity in Naive Slices

**Figure 1:**
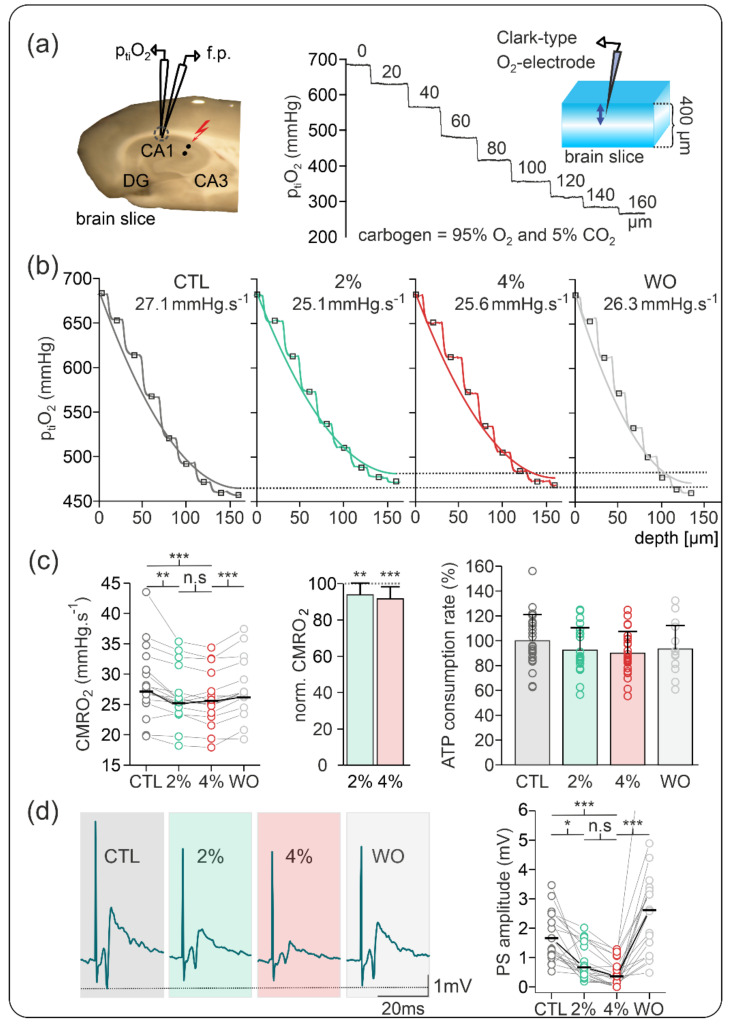
Sevoflurane-induced changes on cerebral metabolic rate of oxygen (CMRO_2_) and synaptic activity in naive slices. (**a**) Representation of recording settings for simultaneous measurements of partial tissue oxygen pressure (p_ti_O_2_) and stimulus-induced population spikes (PSs). Left: Field potential (f.p.) electrode and Clark-style oxygen electrode (p_ti_O_2_) were positioned in the stratum pyramidale of area CA1 while a stimulation electrode (black dots) was placed in the stratum radiatum in area CA2. Right: Exemplary recording of p_ti_O_2_ depth profile in slices gassed with carbogen in an interface condition. Under these conditions, the p_ti_O_2_ of approximately 682 mmHg at the surface of the slices decayed until the lowest oxygen values at the core of the slice. (**b**) Exemplary p_ti_O_2_ depth profiles and CMRO_2_ values as calculated using a reaction–diffusion model in the different conditions: control (CTL, black), under 2% sevoflurane (green), under 4% sevoflurane (red), and after washout (WO, grey). (**c**) Plots of recorded absolute CMRO_2_ values (left) and normalized CMRO_2_ changes (middle) showing a small but significant decrease under 2% sevoflurane, no further significant changes under 4% sevoflurane, and reversibility. Based on the experimental data, the computed relative adenosine triphosphate (ATP) consumption rate slightly decreased in the presence of sevoflurane (right). (**d**) Effects of sevoflurane on stimulus-induced PSs in area CA1. Left: Example traces of PSs in the control condition (dark grey background), under 2% and 4% sevoflurane (green and red backgrounds, respectively) and after washout (grey background). Right: Plot of absolute PS amplitude showing a concentration-dependent decrease under 2% and 4% sevoflurane. After washout, PSs increased, suggesting good reversibility and synaptic facilitation. All point line charts display the median in addition to the absolute values. The bar charts display mean + standard deviation (SD). Statistical comparison: repeated measures ANOVA and Bonferroni post hoc test. * = *p* < 0.05, ** = *p* < 0.01, *** = *p* < 0.001, n.s = *p* > 0.05.

To screen effects of 2% and 4% sevoflurane (i.e., 1 and 2 MAC) on neuronal energy metabolism and synaptic activity, we measured changes in p_ti_O_2_ gradients and stimulus-induced population spikes (PSs) in the stratum pyramidale of the CA1 in naive slices. Based on the recorded p_ti_O_2_ gradients, we calculated changes in the CMRO_2_ and ATP consumption rate using a computational reaction–diffusion model as previously described [34] (Figure 1).

In naive slices, application of sevoflurane slightly decreased CMRO_2_ from 27.13 (23.74, 30.38) mmHg·s^−1^ under control conditions to 25.21 (22.86, 27.28) mmHg·s^−1^ under 2% sevoflurane and to 25.50 (21.99, 27.17) mmHg·s^−1^ under 4% sevoflurane (vs. control *p*  =  0.004 and *p* < 0.001, respectively, *n*  =  20). Normalized CMRO_2_ showed a small decay of −6.2% and −8.4% for 2% and 4% sevoflurane, respectively, suggesting a small concentration-dependent effect. These CMRO_2_ changes corresponded to a reduction in the ATP consumption rate of 7.7% under 2% and 10% under 4% sevoflurane, respectively.

Simultaneously measured stimulus-induced PSs decreased in amplitude from 1.66 (1.11, 2.49) mV in the control to 0.67 (0.42, 1.67) mV under 2% sevoflurane and to 0.36 (0.13, 0.69) mV under 4% sevoflurane (vs. control *p* = 0.004 and *p* < 0.001, respectively *n* = 20). Normalized data revealed a decrease to 58.7% and 47.1% of control PS-amplitude under 2% and 4% sevoflurane, respectively.

Since network activity and metabolism are low in slices under naive conditions [34], we asked whether the effects of sevoflurane in CMRO_2_ are proportional to the neuronal activity and metabolic demand. For this purpose, we studied the effects of sevoflurane during pharmacologically induced gamma oscillations and the subsequent increase in energy demand.

### 2.2. Sevoflurane Effects on Gamma Oscillations and Associated Changes in CMRO_2_

**Figure 2:**
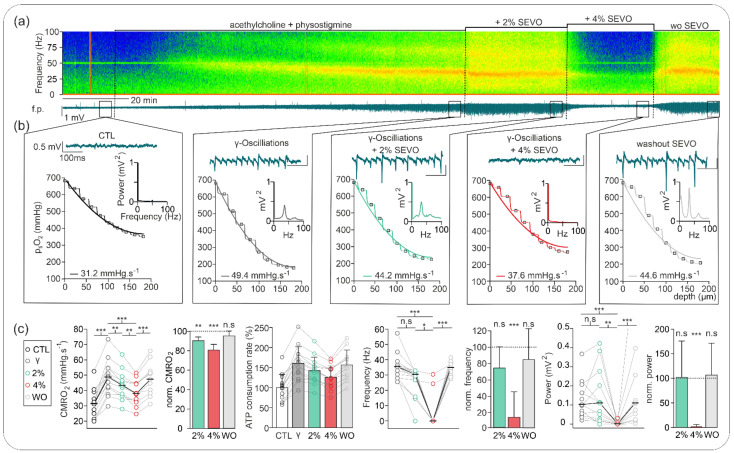
Sevoflurane effects on gamma oscillations and changes in CMRO_2_. (**a**) Exemplary recording of gamma oscillation induction with acetylcholine and physostigmine with subsequent treatment with 2% and 4% sevoflurane and washout (top: online spectrogram, bottom: corresponding f.p. trace). (**b**) Details of recorded network activity and simultaneous changes in p_ti_O_2_ of (**a**), corresponding power spectrum analysis, and calculated CMRO_2_ for each experimental condition of (**a**) (control: black; induced gammas: grey; gamma oscillations under 2% isoflurane: green; gammas under 4%: red; sevoflurane washout: grey). (**c**) Calculated absolute and normalized CMRO_2_ and modeled relative ATP consumption rate showing high energy demand during gamma oscillations. CMRO_2_ significantly decreased under 2% and 4% sevoflurane. After washout, CMRO_2_ was higher than before the induction of gamma oscillations. Concerning gamma oscillations: under 2% sevoflurane, the frequency decreased insignificantly while the power remained unchanged or even slightly increased. Under 4% sevoflurane, gammas were abolished in almost all experiments. After washout effects were reversible. All point line charts display the median in addition to the absolute values. The bar charts display mean +SD. Statistical comparison: repeated measures ANOVA for the CMRO_2_, Friedman test for the gamma oscillations (power and frequency). After Bonferroni correction significance given with * = *p* < 0.05, ** = *p* < 0.01, *** = *p* < 0.001, n.s = *p* > 0.05.

We further studied the effects of sevoflurane on the network activity and metabolic demand during gamma oscillations pharmacologically induced with acetylcholine and physostigmine (Figure 2). This model mimics the cholinergic input from the medial septum into hippocampal neurons. Under these conditions, network oscillations are generated due to simultaneous phasic inhibition and excitation [35]. Particularly, gamma oscillations have been implicated in higher cognitive processes, such as memory processing, and were associated with a marked increase in oxidative metabolism as well [17,36,37,38]. In line with these previous reports, gamma oscillations were related to significant increase in energy demand as CMRO_2_ rose from 31.43 (24.94, 34.52) mmHg·s^−1^ in the naive condition to 48.70 (41.66, 54.50) mmHg·s^−1^ after induction of gamma activity (*p* < 0.001, *n* = 13).

The treatment with 2% sevoflurane generated a clear but nonsignificant decrease in gamma-band frequency from 35.40 (28.08, 36.62) Hz in the control to 30.52 (26.86, 31.74) Hz (*p* = 0.08, *n* = 13), which represented a normalized frequency decrease to 73.6% of control. Under these conditions, changes in power spectrum were not significant (control: 102.0 (55.0, 164.0) µV^2^; under 2% sevoflurane: 110.0 (28.0, 202.0) µV^2^ (*p* = 1.0, *n* = 13)) due to heterogeneous effects (60% of slices with decrease and 40% of slices with increase in gamma power). Application of 4% sevoflurane completely blocked network oscillations in 11 of 13 slices (see analysis in Figure 2).

For 2% and 4% sevoflurane, CMRO_2_ significantly decreased from 48.70 (41.66, 54.50) mmHg·s^−1^ to 43.25 (37.33, 46.49) mmHg·s^−1^ and to 37.94 (32.85, 44.30) mmHg·s^−1^, respectively (vs. control, *p* = 0.001, *n* = 13). Thus, normalized CMRO_2_ decreased in a concentration-dependent manner to 85.1% and to 71.4% of control under 2% and 4% sevoflurane, respectively. Corresponding ATP consumption rate decreased by 12% and 24% under 2% and 4% sevoflurane, respectively.

Importantly, sevoflurane-induced changes in oscillatory activity and CMRO_2_ were completely reversible after a 20 min washout. Accordingly, gamma-band oscillation was restored and showed no significant difference in power, frequency, or CMRO_2_ with the values before treatment (power: 110.0 (54.0, 184.0) µV^2^ (*p* = 1.0, *n* = 13); frequency: 34.84 (32.33, 37.84) Hz (*p* = 1.0); CMRO_2_: 47.37 (40.39, 52.74) mmHg·s^−1^ (*p* vs. control = 0.11)).

Compared to the naive condition, increased cholinergic input during pharmacologically induced gamma oscillations represents a state of highly synchronized neuronal firing with continuously increased energy demand [34]. To further study sevoflurane-induced changes in the energy demand and synaptic activity, we performed measurements of basal CMRO_2_ and stimulus-induced CMRO_2_ in the same experimental protocol.

### 2.3. Effects of Sevoflurane during Stimulus-Induced Energy Demand 

**Figure 3:**
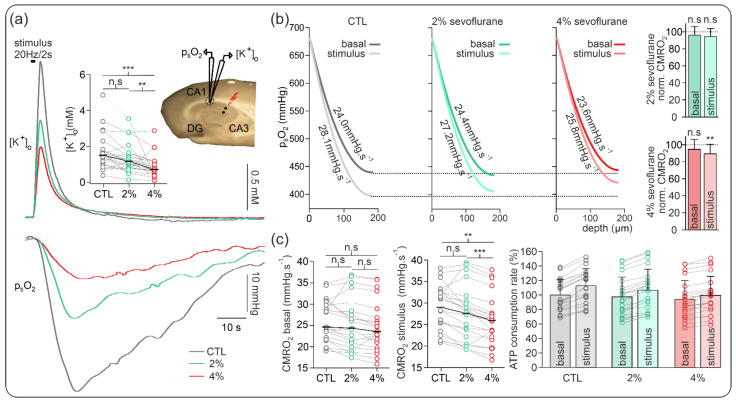
Sevoflurane-induced changes in CMRO_2_ and synaptic activity during electrical stimulation. (**a**) Exemplary recording of simultaneous stimulus-induced (20 Hz, 2 s) p_ti_O_2_ and extracellular potassium ([K^+^]_o_) increases during control conditions (grey) and under sevoflurane treatment (2%: green, 4%: red). Plot of absolute change in stimulus-induced [K_o_]^+^ increases and representation of recording settings. (**b**) Plots of the basal and stimulus-induced changes in CMRO_2_ under control (grey-black), 2% sevoflurane (green), and 4% sevoflurane (red) and normalized changes. The normalized CMRO_2_ data show no significant changes in the basal oxygen consumption under sevoflurane treatment and a significant decrease in the stimulus-induced oxygen consumption under 4% sevoflurane. (**c**) Absolute basal and stimulus-induced CMRO_2_ changes and relative ATP consumption rate. As the basal CMRO_2_ and ATP consumption remained similar, the stimulus-induced changes in metabolism decreased significantly under 4% sevoflurane. All point line charts display the median in addition to the absolute values. The bar charts display mean +SD. Statistical comparison: repeated measures ANOVA for CMRO_2_ and Friedman test for the [K^+^]_o_. After Bonferroni correction significance given with ** = *p* < 0.01, *** = *p* < 0.001, n.s = *p* > 0.05.

We simultaneously measured changes in basal and activity-dependent CMRO_2_ while monitoring stimulus-induced extracellular potassium ([K^+^]_o_) as a correlate of neuronal activation. Sudden increases in energy demand and neuronal activity were elicited by repeated electrical stimulation of the Schaffer collaterals (2 s long 20 Hz tetani, see Section 4 and Figure 3).

Although less pronounced than in our experiments in naive slices, changes in basal CMRO_2_ were marginal, and normalized basal CMRO_2_ decrease was −2.7% and −5.3% of control signal for 2% and 4% sevoflurane, respectively (*n* = 21, see Figure 3).

Concerning stimulus-induced responses, CMRO_2_ and elicited [K^+^]_o_ nonsignificantly decreased from 30.92 (25.32, 32.62) mmHg·s^−1^ in the control to 27.84 (22.16, 30.45) mmHg·s^−1^ (normalized decrease of −5.4% of control, *p* = 0.10, *n* = 21) and from 1.5 (1.01, 1.83) mM to 1.17 (0.77, 1.62) mM (−19.4% of control signal, *p* = 0.07, *n* = 22), respectively. Subsequent treatment with 4% sevoflurane generated a significant decrease in CMRO_2_ to 25.80 (22.16, 27.66) mmHg·s^−1^ (−11.6% of control signal, *p* = 0.002, *n* = 21) and in [K^+^]_o_ to 0.71 (0.45, 1.15) mM (−48.3% of control signal, *p* < 0.001, *n* = 21). Accordingly, the normalized differences between basal CMRO_2_ and stimulus-induced CMRO_2_ for each experimental condition decreased by 28.0% and 41.6% under 2% and 4% sevoflurane, respectively.

The relative changes in ATP consumption rate revealed little changes in basal metabolism (2.6% under 2% and 6.4% under 4% sevoflurane). However, during tetanic electrical stimulation, maximal ATP use decreased by 5.5% and 11.4% under 2% and 4% sevoflurane, respectively.

As measured during increased energy demand (i.e., gamma oscillations and tetanic stimulation), the effects on CMRO_2_ were significant under 4% sevoflurane, i.e., amounts clinically relevant for deep anesthesia. To clarify possible direct effects of high-concentration sevoflurane in mitochondrial function, we performed simultaneous measurements of stimulus-induced changes in FAD and [K^+^]_o_.

### 2.4. Sevoflurane Effects on the FAD Redox State

To determine the cause and effect of the simultaneously reduced CMRO_2_ and neurotransmission, we studied the effect of 4% sevoflurane on FAD redox state at baseline (basal mitochondrial redox state) and stimulus-induced FAD transients evoked by 2 s 20 Hz stimuli. Neuronal excitability state and synaptic input were assessed by simultaneous recordings of [K^+^]_o_ increases and extracellular calcium ([Ca^2+^]_o_) internalization during electrical stimulation. 

**Figure 4:**
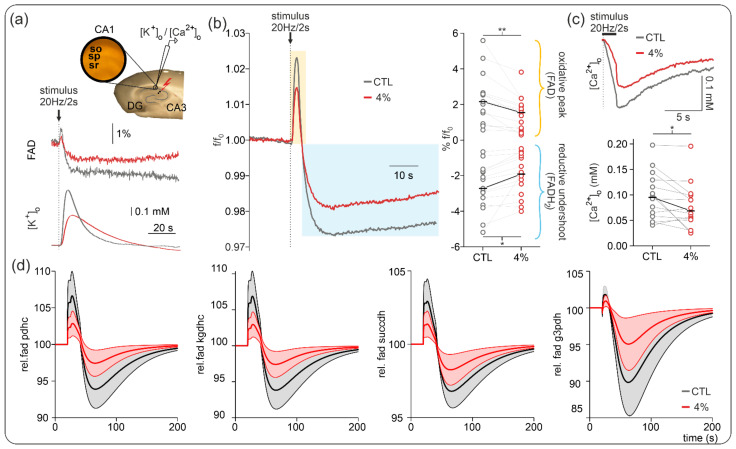
Effects of sevoflurane on flavin adenine dinucleotides (FAD). (**a**) Simultaneous recordings of stimulus-induced changes in FAD autofluorescence and [K^+^]_o_ or [Ca^2+^]_o_ were performed in area CA1 as represented along with example traces of FAD/[K^+^]_o_ in control (black) and under 4% sevoflurane (red). Note that the decrease in FAD peak and undershoot correlated with decreased [K^+^]_o_ rises as tissue excitability decreases as well. (**b**) Left: Averaged stimulus-induced FAD transients in control (black trace) and under 4% sevoflurane (red trace). Stimulus-induced FAD signals typically have two components: a first oxidative peak immediately after stimulation followed by a reductive undershoot. In the presence of 4% sevoflurane, both components diminished as synaptic activity decreased. (**c**) Ca^2+^ input onto neurons decreased under 4% sevoflurane, generating the boundary conditions for an activity-dependent reduction in oxidative metabolism. Statistical comparison: repeated measures ANOVA. After Bonferroni correction significance given with * = *p* < 0.05, ** = *p* < 0.01. (**d**) Modeling of relative changes in FAD signaling during electrical stimulation without sevoflurane (black) and with 4% isoflurane (red) for the pyruvate dehydrogenase (pdhc), α-ketoglutarate dehydrogenase (kgdhc), succinate dehydrogenase (succdh), and mitochondrial glycerol-3-phosphate dehydrogenase (g3pdh). The solid line and shaded area depict the mean and SD of simulated FAD signals for the individual slices.

As shown in Figure 4, 4% sevoflurane significantly decreased both components of the stimulus-induced FAD transients (i.e., oxidative peak and reductive undershoot). Accordingly, the oxidative peak decreased from 2.16 (1.53, 2.66) under control conditions to 1.55 (0.68, 1.97), and the reductive undershoot diminished from −2.72 (−3.24, −1.76) under control conditions to −1.92 (−2.47, −0.89) (fluorescence calculated as f/f_0_, *p* = 0.005 and *p* = 0.013, respectively, *n* = 15).

As the intensity of the FAD fluorescence signal decreases continuously due to photodecomposition of the flavin chromophore [39], changes in bleaching behavior might reveal alterations in the redox state of enzymes containing FAD as an electron donor [39,40]. Analysis of the fluorescence decay revealed no significant changes in basal redox state in the presence of 4% sevoflurane (f/f_0_ control: 0.54 (0.33, 0.56), f/f_0_ sevoflurane 4%: 0.47 (0.44, 0.74), *p* = 0.70, *n* = 8) indicating that sevoflurane effects on basal energy metabolism were marginal.

### 2.5. Computational Modeling: Effects on Mitochondrial FAD during Electrical Stimulation

In our experiments, changes in CMRO_2_ reflected changes in energy demand due to inhibition of electrophysiological processes suppressed by sevoflurane application. Additionally, there might be a direct effect of sevoflurane on mitochondrial processes that could limit energy production rate and thereby limit CMRO_2_, independently from electrophysiological inhibition. If so, this would be reflected in changes in mitochondrial redox potential as measured by FAD autofluorescence [17,38,41,42].

To check whether the induced changes in CMRO_2_ are consistent with FAD fluorescence changes, without the assumption of an additional inhibition of mitochondrial enzymes, we used a computational model of neuronal energy metabolism [40] to simulate FAD fluorescence changes during rest and electrical stimulation with and without sevoflurane application. Glucose and O_2_ were assumed to be constant and sufficient, corresponding to constant bath perfusion of artificial cerebrospinal fluid (aCSF) with 10 mM glucose and oxygenation with 95% oxygen. Since there are no differences in the contribution of different layers of the slice when O_2_ availability is not limited, we used a homogeneous model as in the work of Berndt et al. (2020) [40].

Metabolic demand was adjusted for each slice and each condition to match the measured maximal CMRO_2_. Next, for each slice, electrical stimulation was simulated by a steep increase in ATP demand and a corresponding influx of Ca^2+^ as measured in our experiments (see also [17,38]). The simulated FAD redox state was then compared to the measured FAD fluorescence for each slice.

Figure 4 shows that the decrease in CMRO_2_ during the application of 4% sevoflurane is in agreement with the modeled changes in the oxidative peak and the reductive undershoot of the stimulus-induced FAD signal and state of the FAD-containing mitochondrial enzymes pyruvate dehydrogenase (PDHC), α-ketoglutarate dehydrogenase (KGDHC), and succinate dehydrogenase (SUCCDH). The stimulus-induced increase in energy demand led to a decrease in cytosolic ATP, which activated the nucleotide translocator exchanging mitochondrial ATP with cytosolic ADP, thereby decreasing mitochondrial ATP. The decrease in mitochondrial ATP leads to stimulation of the respiratory chain and oxidative phosphorylation, which leads to increased ATP production and increased O_2_ consumption. To compensate for the resulting decrease in mitochondrial NADH redox state, the citric acid cycle was activated partially by the reduced NADH redox state and partially by Ca^2+^ influx, resulting in the observed oxidative shift in the FAD-containing enzymes. Once the ATP demand decreased to resting values after electrical stimulation was over, the persisting activation of the citric acid cycle by Ca^2+^ resulted in a reductive shift in the FAD-containing enzymes and the observed undershoot. Only the mitochondrial glycerol-3-phosphate dehydrogenase (G3PDH) shows a negligible overshoot and a very pronounced undershoot. This is because it is coupled to the cytosolic NADH redox potential, which becomes reduced due to increased glycolytic activity following energetic stimulation [42].

## 3. Discussion

To investigate the impact of clinically relevant concentrations of sevoflurane on oxidative metabolism in neuronal tissue, we studied the effect of 2% and 4% sevoflurane (i.e., 1 and 2 MAC) on electrophysiological signals and CMRO_2_ during different activity patterns in brain slices (i.e., naive slices, during gamma oscillations, and electrical stimulation).

### 3.1. Effects of Sevoflurane on Neurometabolism Depend on Neuronal Activity States

Sevoflurane-induced decrease in normalized CMRO_2_ was marginal in naive slices (6.2% and 8.4% for 2% and 4% sevoflurane, respectively). Under these conditions, the field potential PSs decreased in a concentration-dependent manner (60% and 78% of control for 2% and 4% sevoflurane, respectively). These results suggest that sevoflurane effects in neuronal respiration were small because global spontaneous activity in naive slices is low. As stimulus-induced synaptic transmission (single pulse PSs) decreased in amplitude, changes in electrophysiological processes and related decrease in energy demand occurred with high efficiency. However, as changes in electrically induced single PSs do not necessarily correlate with the metabolic state of the tissue [36], we further studied sevoflurane effects during increased neuronal network activity.

Thus, to investigate the possible interdependency between effects on CMRO_2_ and neuronal activity, we tested sevoflurane on pharmacologically induced gamma oscillations and during prolonged stimulus-induced neuronal activation (Figure 2 and Figure 3, respectively). 

Gamma oscillations are generated by synchronous cell firing, which is the result of rhythmic perisomatic GABAergic inhibition of pyramidal cells [43]. In the preparation of slices, optimal recruitment of the vast majority of neurons in the network occurs during gamma oscillations, and sustained energy demand increases [34]. Indeed, mitochondrial oxidative capacity might work near the limit during in vitro gamma oscillations [37]. The treatment with 2% sevoflurane decreased CMRO_2_, while slightly reducing gamma frequency with heterogeneous effects on oscillatory power (Figure 2). Subsequent application of 4% sevoflurane almost completely abolished gamma oscillations. The moderate CMRO_2_ decrease at 2% sevoflurane (15%) correlated with lower oscillatory frequencies since increased inhibitory input lowered the firing rate of pyramidal neurons [44]. The application of 4% sevoflurane abolished network oscillations and decreased CMRO_2_ by about 30%. Importantly, CMRO_2_ under 4% sevoflurane was higher than that before gamma induction (control gamma oscillations), and abolishment was completely reversible, demonstrating indirectly that oxidative metabolism remained intact even at and after concentrations relevant for deep anesthesia. 

To confirm that sevoflurane-induced decrease in CMRO_2_ was highly coupled to neuronal activity, we simultaneously measured changes in basal CMRO_2_ and during stimulus-induced neuronal activation during low and high energy demand in the same experimental protocol (Figure 3). In line with our experiments in naive slices, basal CMRO_2_ was minimally decreased by 2% and 4% sevoflurane. Corroborating our results on network oscillations as well, stimulus-induced CMRO_2_ decreased to 85.1% and 71.4% of control, as did the monitored increases in [K^+^]_o_. In line with our previous results, 4% sevoflurane, i.e., amounts relevant for deep anesthesia, significantly reduced neuronal activity and metabolism.

### 3.2. No Evidence for Direct Impairment of the Mitochondrial Enzymes at Clinically Used Sevoflurane Concentrations

Mitochondrial dysfunction has been previously described for sevoflurane in experimental studies [14,45,46]. To clarify whether the CMRO_2_ decrease by 4% sevoflurane might be related to mitochondrial impairment, we performed FAD imaging to monitor mitochondrial redox state while measuring [K^+^]_o_ as a correlate for neuronal activity and [Ca^2+^]_o_ as the main trigger for intracellular metabolic activation [47]. FAD (oxidized) contains isoalloxazine chromophore, which is fluorescent when excited with blue light in the oxidized form, while reduced FADH_2_ is not fluorescent [39,40,41]. Changes in the redox state of FAD (baseline and stimulus-induced FAD transients) are mainly caused by redox reactions of the mitochondrial PDHC, KGDHC, G3PDH, and SUCCDH complexes [42]. As shown in Figure 4, 4% sevoflurane significantly decreased both components of the stimulus-induced FAD transients (i.e., oxidative peak and reductive undershoot) without changes in the FAD baseline. Integrating our experimental data with modeled FAD redox states, we did not find evidence for direct inhibition of mitochondrial enzymes by sevoflurane. Rather, the reduced ATP consumption due to sevoflurane-induced electrophysiological inhibition leads to decreased activation of the respiratory chain due to decreased Ca^2+^ influx.

### 3.3. Metabolic Profile of Sevoflurane and Translational Relevance

Since 2% and 4% sevoflurane represent 1 and 2 MAC, our results are relevant for the states of light and deep general anesthesia, respectively. Our explorative study suggests that during sevoflurane-induced general anesthesia with 1 MAC, changes in energy homeostasis are almost negligible. Since intracellular ATP production would not decrease due to hypometabolism, situations with low flow of oxygen and/or substrate (i.e., intraoperative low blood pressure) would not potentially generate an energy mismatch between supply and demand. In line with our results, CMRO_2_ remained stable or slightly decreased in patients treated with 1 MAC sevoflurane [48,49]. However, measurements in vivo (in humans or animals) are challenging since changes in CMRO_2_ and cerebral blood flow are interdependent [50]. For this reason, we took advantage of an in vitro preparation with a constant supply of oxygen and glucose. 

Only 4% sevoflurane generated strong changes in neuronal respiration, which is a concentration relevant for the induction of deep anesthesia. Although controversial, burst suppression induction with high-dose anesthetics is a clinical strategy used to decrease cerebral metabolism in critical situations, such as status epilepticus and intracranial hypertension [8,51]. As shown in our experiments concerning gamma oscillations and stimulus-induced energy demand, high-concentration sevoflurane reliably and reversely decreased CMRO_2_ by inhibiting neuronal activity. On the other hand, the poor effect of high-dose sevoflurane in states of low basal metabolism (naive and stimulated slices [36]) suggested no benefit in situations with impaired neuronal activity (i.e., intoxication or hypothermia). For these reasons, anesthesia has to be conducted while monitoring electroencephalography to avoid unnecessary overdosing by using standard MAC-values.

### 3.4. Isoflurane vs. Sevoflurane

Since multiple anesthetics are available, it is important to compare available options on the different aspects of brain function. In previous work, we used methods similar to those described here to investigate the effect of isoflurane on neuronal energy metabolism and neuronal electrophysiology [38].

Although isoflurane and sevoflurane belong to the same class of volatile general anesthetics, several publications have described these two drugs as being selective in their neurophysiologic effects. Therefore, isoflurane and sevoflurane modulate synaptic input onto hippocampal neurons and interneurons in an agent-specific manner [19,52]. 

Comparing sevoflurane and isoflurane effects at clinically relevant concentrations, isoflurane had a stronger effect on synaptic processes and oxidative metabolism. For example, PS amplitude was twice as big under 2 MAC (4%) sevoflurane than under 2 MAC (3%) isoflurane (23.52% vs. 11.30% from their respective control amplitudes) [38]. 

During gamma oscillations, both volatile anesthetics diminished the frequency in a similar amount (1 MAC isoflurane: −9.35%; 1 MAC sevoflurane: −13.87%, compared to their respective control conditions). However, while 1 MAC sevoflurane did not impact the oscillation’s power (+10.0% compared to control condition), 1 MAC isoflurane reduced it by 46.62%, severely compromising the synchrony of the oscillations [38]. 

Mitochondrial dysfunction has been described for both anesthetics using multiple experimental approaches [12,30,32,45,53,54,55]. In particular, direct inhibition of mitochondrial complexes I, II, and III by volatile anesthetics have been involved in presynaptic modulation, mitochondrial depolarization, decreased ATP production, and preconditioning [13,14,56,57]. The majority of these studies were performed in isolated preparations and different species (isolated mitochondria, synaptosomes, or *C. elegans*), and the performed measurements focused on changes in mitochondrial membrane potential and molecular biology methods. In our approach, integrating electrophysiological data, oxygen recordings, FAD imaging, and theoretical modeling, we did not find evidence for direct inhibition of neuronal energy metabolism during short application of either drug at clinically relevant dosages. Importantly, metabolic effects were in general stronger when using isoflurane than when using sevoflurane. For example, while 2 MAC sevoflurane decreased basal CMRO_2_ by only 5%, the effect was twice as strong for 2 MAC isoflurane [38]. Thus, a secondary decrease in ATP production with a potential energetic mismatch in the case of a sudden increase in metabolic demand (i.e., during seizures) might be less probable under sevoflurane. In consequence, the use of sevoflurane could have some advantages in achieving neuroprotection in certain clinical situations such as super-refractory status epilepticus, which might be treated with isoflurane [8]. For these reasons, more research is necessary to differentiate the best pharmacological profile between both inhalation anesthetics.

### 3.5. Isoflurane and Sevoflurane vs. Propofol

In line with in vivo studies [15], we have previously shown that propofol, unlike sevoflurane and isoflurane, alters the neuronal function not only by blocking electrophysiological processes in neuronal tissue but also due to direct inhibition of the complex II of the respiratory chain [17]. While the experimental concentrations of isoflurane and sevoflurane can be related to clinically used concentrations, this is not possible for propofol under similar experimental conditions. Although the used propofol concentrations elicited similar electrophysiological changes, the concentrations were far higher than the clinically used concentrations.

## 4. Materials and Methods

This study complies with the ARRIVE 2.0 guidelines, the Helsinki declaration, and the Charité animal welfare guidelines. The experimental protocols were approved by the State Office of Health and Social Affairs of Berlin (T0096/02). Before tissue extraction for slice experiments, the animals had at least 7 days for acclimation in our animal shed. The accommodation was in groups of two with food ad libitum and a 12 h light on/light off cycle.

### 4.1. Slice Preparation and Maintenance

For in vitro experiments, hippocampal slices were prepared from 27 young male Wistar rats (weight: 200 g, age: 8  ±  1 weeks) as previously described [10]. ACSF contained (in mM): 129 NaCl, 21 NaHCO_3_, 10 glucose, 3 KCl, 1.25 NaH_2_PO_4_, 1.6 CaCl_2_, and 1.2 MgCl_2_. Osmolarity was 295–305 mosmol/L and pH was 7.35–7.45.

### 4.2. Electrophysiology, p_ti_O_2_ Recordings, and Fluorescence Imaging

Recordings were performed in the stratum pyramidale in area CA1 while electrical stimulation with a bipolar platinum electrode (20 µm) was applied in stratum radiatum in area CA2. To monitor changes in synaptic transmission, single pulses (100 µs duration, 50 ms interval) were applied at an intensity to evoke 75% of maximal amplitude. As previously reported [10,38,39], sudden and strong neuronal activation was induced by 2 s long 20 Hz tetani (single pulse duration 100 µs, pulse interval 50 ms, 40 pulses, tetani interval 3 min) to study activity-dependent changes in p_ti_O_2_, associated [K^+^]_o_ increase, and [Ca^2+^]_o_ decrease. The repeated application of 2 s long 20 Hz stimuli generated stable electrophysiological and metabolic signals, which are suitable for stable recording conditions (see also [10,39]). Extracellular ion changes were measured using double-barreled ion-sensitive microelectrodes constructed and calibrated as reported [10]. Simultaneous FAD and [K^+^]_o_ recordings were performed to study changes in baseline mitochondrial redox state during neuronal activation (i.e., during 2 s long, 20 Hz tetani). To measure changes in p_ti_O_2_ and perform CMRO_2_ calculations, the O_2_ electrode was moved vertically through the slice in 20 μm steps until reaching the minimum of ptiO_2_. During tetanic stimulation, 20 µm steps were performed as usual, and stimulus-induced ptiO_2_ decreases were measured during three stimuli at defined depths (40 µm, 120 µm, and core). CMRO_2_ calculations were performed offline as described below. FAD autofluorescence imaging was performed in area CA1 with a 20x objective (numerical aperture 0.5) using a custom-built setup equipped with a light-emitting diode (LED, 460 nm wavelength, Lumen, Prior Scientific, Seefelder, Germany) and a photomultiplier tube (PMT, Seefelder, Messtechnik, Germany). To reduce bleaching and phototoxicity, light pulses at 5 Hz (5 s duration) were applied as previously described [39].

### 4.3. Sevoflurane Application and Induction of Gamma Oscillations

Sevoflurane in concentrations of 2% or 4% (i.e., 1 or 2 MAC, respectively) was applied for 20 min (each concentration) in an interface system or dissolved in aCSF (for FAD imaging in submerged condition) together with carbogen (95% O_2_ and 5% CO_2_) with a calibrated Sevoflurane vaporizer (Dräger, Lübeck, Germany). The concentration of sevoflurane was controlled using a Vamos mobile sevoflurane monitor (Dräger, Lübeck, Germany). The recording chamber temperature was 36 °C for both the interface and submerged conditions. Taking into account a water/gas partition coefficient for 36 °C of 0.4, the application of 2% and 4% correspond to 0.3 and 0.6 mM sevoflurane in the aCSF [58]. Gamma oscillations were induced by the application of 10 µM acetylcholine chloride and 2 µM physostigmine salicylate (Sigma-Aldrich, Steinheim, Germany).

### 4.4. Data Acquisition and Data Analysis

Analog signals were digitalized with a Power CED1401 and Spike2 software (Cambridge Electronic Design, Cambridge, UK). Data analysis and statistics were performed using Spike2, Excel (Microsoft, Seattle, WA, USA), Origin (Version 6, Microcal Software, Northampton, MA, USA), and SPSS (Chicago, IL, USA). The median values and corresponding 25th and 75th percentiles in brackets are described in Section 2. Data are shown in box plots (with median, mean, and 25th and 75th percentiles) or dot plots with corresponding medians or means ± standard deviation as described. Fluorescence of FAD is shown as Δf/f_0_ where f_0_ is the average of 15 s baseline before stimulation. Fluorescence decay was analyzed by normalization of 3 min before stimulation in the control, under treatment, and after washout of sevoflurane (f_0_ was the measured first point). For statistical comparison, the percent decay after 3 min was compared. Power spectra of gamma oscillations were calculated with Spike2 (5 min per condition, FFT, Hanning window, size 4096). Statistical analysis of absolute data was performed using SPSS software. The Shapiro–Wilk normality test was used to test the Gaussian distribution of the variables. Subsequently, repeated measures ANOVA or Friedman tests were used according to the normality of the data. For comparison and legibility, mean percent changes (internal normalization to control for each experiment) were calculated. Changes were stipulated to be significant for *p* values < 0.05 after Bonferroni correction. No outliers were removed from the data.

### 4.5. Calculation of Cerebral Metabolic Rate of O_2_

As previously described, CMRO_2_ was calculated from p_ti_O_2_ depth profiles [17]. In short, we applied a reaction–diffusion model for O_2_ consisting of diffusive O_2_-transport and O_2_-consumption within the slice. Slices were divided into layers with an equal thickness of 1 μm. The diffusive distribution of O_2_ between the layers is described by Fick’s law with a diffusion constant of 1.6 × 103 μm^2^/s, and the O_2_ consumption rate within each layer is given by Michaelis–Menten kinetics with a K_m_ value of 3 mmHg [59]. The CMRO_2_ was assumed to be homogeneous throughout the slice and was treated as an adjustable parameter to match the experimental data. For the boundary conditions, the p_ti_O_2_ concentration at the slice surface was fixed to the supply value, while at the p_ti_O_2_ minimum, the diffusive transport of O_2_ was put to zero.

### 4.6. Calculations of FAD Transients and ATP Consumption Rates

As alterations in FAD fluorescence originate from the PDHC, KGDHC, G3PDH, and SUCCDH complexes, fluorometric measurements of FAD permit the study of the mitochondrial redox state [41,42]. Based on calculated CMRO_2_, we used the metabolic model of neuronal energy metabolism to simulate stimulus-induced FAD transients and ATP consumption rates as established and described by Berndt et al. (2015) [42]. Differences in basal CMRO_2_ during sevoflurane administration imply differences in basal ATP consumption rates, as increased ATP consumption lowers ATP levels; activates glycolysis, citric acid cycle, and respiratory chain activity; and concomitantly increases CMRO_2_. Sevoflurane-induced changes in basal ATP consumption rates were simulated by adaptation of the resting ATP demand to match the observed resting CMRO_2_. In experiments with electrical stimulation, we simulated the time-dependent metabolic response to a brief stimulus-induced increased ATP demand and corresponding cytosolic Ca^2+^ transient in addition to the sevoflurane-dependent changes in the metabolic resting state. The time course of the energetic load, i.e., the increase in the ATP demand associated with the activating stimulus, was described by a rectangular activation function describing a short period of high metabolic demand (corresponding to the duration of stimulation), while the associated cytosolic Ca^2+^ transient was modeled as steep Ca^2+^ increase (corresponding to sudden stimulus-induced Ca^2+^ influx into the cell) followed by a slowly decaying component (corresponding to the slower pumping of Ca^2+^ from the cytosol out of the cell). The magnitude of the stimulus was set by taking into account the calculated CMRO_2_ in control and after 4% sevoflurane. Cytosolic Ca^2+^ is rapidly taken up into mitochondria by Ca^2+^ uniporter. The Ca^2+^ taken up by the mitochondria is first sequestered by Ca^2+^-binding proteins and then released into the mitochondrial matrix where it activates the mitochondrial PDHC, isocitrate dehydrogenase, and KGDHC. At the same time, increased ATP demand decreases cytosolic ATP levels, thereby activating glycolysis and cellular shuttle systems and increasing mitochondrial activity. The corresponding changes in the reduction state of protein-bound FAD moieties were then compared to the observed FAD fluorescence changes obtained by the fluorometric measurements for validation. For all simulations, we used MATLAB Release 2012a (The MathWorks, Inc., Natick, MA, USA) with the optimization toolbox.

## 5. Conclusions

Only at concentrations clinically relevant for deep anesthesia, sevoflurane significantly decreased neuronal metabolism. Our results suggest that the inhibition of synaptic processes and network activity is the main source of energy demand decrease. Compared to isoflurane, sevoflurane has less pronounced effects on neurometabolism at similar clinically relevant concentrations. Unlike propofol, we did not find that sevoflurane directly impacts mitochondrial enzymes after short-term anesthesia in hippocampal slice preparations of young adult rats. 

## Data Availability

The data that support the findings of this study are available from the corresponding author upon reasonable request.
